# Concerted Action of ANP and Dopamine D1-Receptor to Regulate Sodium Homeostasis in Nephrotic Syndrome

**DOI:** 10.1155/2013/397391

**Published:** 2013-07-15

**Authors:** Cátia Fernandes-Cerqueira, Benedita Sampaio-Maia, Janete Quelhas-Santos, Mónica Moreira-Rodrigues, Liliana Simões-Silva, Ana M. Blazquez-Medela, C. Martinez-Salgado, Jose M. Lopez-Novoa, Manuel Pestana

**Affiliations:** ^1^Nephrology Research & Development Unit, Faculty of Medicine, University of Porto & Hospital S. João EPE, Alameda Prof. Hernâni Monteiro, 4200-319 Porto, Portugal; ^2^Faculty of Dental Medicine, University of Porto, Rua Dr. Manuel Pereira da Silva, 4200-393 Porto, Portugal; ^3^Laboratory of General Physiology, Unit for Multidisciplinary Biomedical Research (UMIB), Institute of Biomedical Sciences Abel Salazar, University of Porto (ICBAS-UP), Rua de Jorge Viterbo Ferreira No. 228, 4050-313 Porto, Portugal; ^4^Renal y Cardiovascular Physiopathology Research Unit, Instituto Reina Sofia de Investigación Nefrológica, Department of Physiology and Pharmacology, University of Salamanca, Edificio Departamental S-20 Campus Miguel de Unamuno, 37007 Salamanca, Spain; ^5^Instituto de Estudios de Ciencias de la Salud de Castilla y León (IECSCYL), Research Unit, University Hospital of Salamanca, Paseo de San Vicente 58-182, 37007 Salamanca, Spain

## Abstract

The edema formation in nephrotic syndrome (NS) is associated with a blunted response to atrial natriuretic peptide (ANP). The natriuretic effects of ANP have been related to renal dopamine D1-receptors (D1R). We examined the interaction between ANP and renal D1R in rats with puromycin aminonucleoside-induced NS (PAN-NS). Urinary sodium, cyclic guanosine monophosphate (cGMP) excretion, and D1R protein expression and localization in renal tubules were evaluated in PAN-NS and control rats before and during volume expansion (VE). The effects of zaprinast (phosphodiesterase type 5 inhibitor), alone or in combination with Sch-23390 (D1R antagonist), were examined in both groups. The increased natriuresis and urinary cGMP excretion evoked by acute VE were blunted in PAN-NS despite increased levels of circulating ANP. This was accompanied in PAN-NS by a marked decrease of D1R expression in the renal tubules. Infusion of zaprinast in PAN-NS resulted in increased urinary excretion of cGMP and sodium to similar levels of control rats and increased expression of D1R in the plasma membrane of renal tubular cells. Combined administration of Sch-23390 and zaprinast prevented natriuresis and increased cGMP excretion induced by zaprinast alone. We conclude that D1R may play a major role in the ANP resistance observed in PAN-NS.

## 1. Introduction 

Nephrotic syndrome (NS) is characterized by increased proteinuria, accompanied by sodium retention that can lead to edema formation and ascites accumulation [1]. Sodium retention in NS was traditionally considered to result from reduced plasma volume associated with reduced serum albumin concentration [1]. However, this hypovolemia concept cannot explain all features of enhanced sodium retention in NS, and a primary intrarenal sodium handling abnormality was also implicated in this condition [2]. This abnormality was attributed to an increase in activity of the Na^+^/H^+^ exchanger (NHE_3_) in the proximal tubules associated with a shift of this transporter from the inactive to an active pool [3] as well as to a blunted response to atrial natriuretic peptide (ANP) [4] and enhanced Na^+^, K^+^-ATPase activity in the cortical collecting duct [5]. The ANP resistance observed after ANP binding to its receptors in cortical collecting duct appears to result from the enhanced activity of phosphodiesterase type 5 (PDE5), an enzyme responsible for the catabolism of cyclic guanosine monophosphate (cGMP), the second messenger of ANP [6, 7]. Dopamine of renal origin is an endogenous natriuretic hormone that plays a central role in sodium homeostasis and blood pressure control [8, 9]. Dopamine formed in proximal tubular cells decreases tubular sodium reabsorption by inhibiting Na^+^, K^+^-ATPase and the NHE_3_ both in the proximal tubule and in more distal segments of the nephron [10, 11]. The natriuretic effects of dopamine mainly result from the activation of dopamine D1R, a G protein-coupled receptor, in renal tubules [12]. Our group has shown previously that rats with puromycin aminonucleoside- (PAN-) induced NS (PAN-NS) show a blunted activity of the renal dopaminergic system evidenced by decreased urine dopamine output and diminished aromatic L-amino acid decarboxylase activity, the enzyme responsible for dopamine synthesis in renal proximal tubules [13]. The finding in PAN-NS rats that the increase in Na^+^, K^+^-ATPase activity in renal proximal tubules was accompanied by blunted natriuresis during D1R agonist fenoldopam infusion, in normal as well as volume expanded conditions [13], suggested that a decreased availability of D1R in renal proximal tubules of PAN-NS might contribute to sodium retention in this situation.

Renal dopamine and ANP are known to interact with each other in order to regulate sodium homeostasis [14–16]. Dopamine and D1R appear to play critical roles in the *in vivo* natriuretic effect of ANP, which inhibits apical NHE_3_ via a dopamine-dependent mechanism [17]. The complex interaction between these two natriuretic systems may be related with the ability of ANP to recruit silent D1R from the interior of the renal tubular cells towards the plasma membrane where they become functionally active [18]. 

The aim of the present study was to examine the interaction between ANP and the renal D1R in the control of sodium homeostasis in PAN-NS. For this purpose, normal and nephrotic rats were subjected to extracellular fluid volume expansion, and the influence of the PDE5 inhibitor zaprinast alone or in combination with the D1R antagonist Sch-23390 on natriuresis, urinary cGMP excretion, and immunolocalization of D1R in renal tubular cells was evaluated. Our results support the hypothesis that D1R may play a major role in the resistance to ANP in PAN-NS.

## 2. Materials and Methods

### 2.1. *In Vivo* Studies

All *in vivo* investigations were performed in accordance with the European Directive number 86/609, transposed to the Portuguese Law by DL 129/92 and by Portaria 1005/92.

*PAN-NS*. Normotensive male Sprague-Dawley rats (Harlan, Barcelona, Spain), weighing ~150 g, were selected after a seven-day period of stabilization. The PAN-NS was induced in rats as previously reported [13].

*Metabolic Studies*. The animals were kept under controlled environmental conditions (12 : 12 h light/dark cycle and room temperature 22 ± 2°C). The PAN-NS and control groups of rats had free access to tap water. In order to achieve the same daily sodium intake in the two groups, the PAN-NS rats were fed *ad libitum* throughout the study with ordinary rat chow (Panlab, Spain) containing 1.9 g/Kg of sodium, whereas the control rats had only access to the mean daily rat chow intake of the PAN-NS animals (Table 1). Two days before sacrifice, the animals were housed in metabolic cages (Techiplast, Italy) in order to collect 24 h urine for determination of urinary excretion of sodium, potassium, creatinine, and proteins. Some animals were sacrificed on day 7 (PAN-NS, *n* = 5; control, *n* = 5) or on day 14 (PAN-NS, *n* = 4; control, *n* = 4) after injection. On the day of sacrifice the animals were anaesthetized with sodium pentobarbital (50 mg/kg bw; ip). Afterwards the ascites volumes were measured, blood was collected for later determination of sodium, potassium, creatinine, proteins, and ANP in plasma and the kidneys were rapidly removed, weighed, and stored as previously reported [13].

*Infusion with PDE5 Inhibitor and D1R Antagonist during Volume Expansion (VE). *For this set of experiments, 7 days after injection, during the phase of maximum sodium retention and ascites accumulation rats were divided into 6 experimental groups: PAN-NS and control rats infused with vehicle 0.9% NaCl (PAN-NS V, *n* = 4; control V, *n* = 3); PAN-NS and control rats infused with the PDE5 inhibitor zaprinast (PAN-NS Z, *n* = 4; control Z, *n* = 3), and PAN-NS and control rats infused with zaprinast plus the D1R antagonist Sch-23390 (PAN-NS ZS, *n* = 4; control ZS, *n* = 3). The animals were surgically manipulated for jugular drug infusion and urine collection as previously reported [13]. The infusion of the vehicle (0.9% NaCl), zaprinast (100 *μ*g/kg bw/min), or zaprinast (100 *μ*g/kg bw/min) plus Sch-23390 (30 *μ*g/kg bw/min) started at a rate of 5 mL/kg bw/h for 120 min; during this period two consecutive 60 min urine samples were collected (*t* = 0–120 min, basal period). At the end of this stabilization period, the VE started with the administration of a volume equal to 5% of the rat's weight over a 30-minute interval (50 mL/kg bw/30 min, *t* = 120–150 min, VE period). Subsequently, the infusion was reduced to the basal rate for another 30 min (*t* = 150–180 min, recovery period, R-VE). Throughout the experiment urine was collected in empty vials for later determination of sodium, creatinine, and cGMP. At the end of the protocol the animals were euthanized and the blood was collected for ANP analysis. Fragments of the left kidney were cut and kept in 4% aqueous formalin for later immunofluorescence procedures.

### 2.2. *In Vitro* Studies

*Western Blotting.* Renal cortex homogenates together with protein extraction reagent supplemented with phosphatases and proteases inhibitors (Pierce, USA) were mixed in loading buffer (0.35 M tris-HCl, 4% SDS, 30% glycerol, 9.3% DTT, pH 6.8, and 0.01% bromophenol blue) and denatured at 95°C for 5 minutes. Total proteins from renal cortex (20 *μ*g per well) were separated by SDS-PAGE on a 10% polyacrylamide gel and then transferred to a nitrocellulose membrane (Bio-Rad Laboratories, USA) at 20 V for 1 h at room temperature with a semidry transfer unit (Bio-Rad). Membranes were incubated with rabbit polyclonal anti-D1R antibody (1/100, Chemicon International Millipore, USA) overnight at 4°C. After being incubated with the secondary antibody, fluorescently labelled donkey anti-rabbit (1/20000, IRDye800, Rockland, USA), the membranes were scanned with the Odyssey Infrared Imaging System (LI-COR Biosciences, USA). The intensity values were normalized for *β*-actin and shown as percentage of control rats.

*Double-Immunofluorescence Staining*. Immunohistochemistry was performed on buffered formalin fixed, paraffin-embedded tissue sections. Briefly, sections were deparaffined in xylene and rehydrated in graded ethanols. Heat-induced antigen retrieval was performed in Tris-EDTA buffer (1 M Tris, 1 mM EDTA, and pH 8.00). Sections were washed with PBS, permeabilized with 0.1% Triton X-100 and 2% fatty-free dehydrated milk, blocked with 2% fatty-free dehydrated milk in PBS for 1 hour, and incubated during 1 hour with goat anti-rat megalin antibody (dilution 1/100; Santa Cruz Biotechnologies, Germany, ref. sc-16478), washed with PBS, and incubated 45 min with anti-goat Alexa fluor 488 (dilution 1/1000, Molecular Probes-Invitrogen, Spain, ref. A-11055). After washing with PBS, sections were then incubated during 1 hour with rabbit anti-rat D1R antibody (1/1000, Chemicon-Millipore Corporation, Spain, ref. AB1784P), washed with PBS and incubated during 45 min with goat anti-rabbit Cy3 (dilution 1/500, Jackson Immunoresearch-Vitro, Spain, ref. 113-165-003). A nucleus staining was performed by 5 min incubation with 2 *μ*M Hoechst 33258 (Molecular Probes) in a dark chamber. Cover slips were mounted on slides using Prolong gold antifade (Molecular Probes). Confocal images were made using a Zeiss Axiovert 200 M microscope and a Zeiss LSM 510 confocal module, with a HeNe laser with 543-excitation for rhodamine, Ar laser with 488-excitation for FITC, and Hg laser with 365-excitation for Hoescht. All images were obtained with identical parameters for intensity, pinhole aperture, and so forth.

*Plasma and Urine Ionogram and Biochemistry*. The quantification of sodium, potassium, total proteins, and creatinine was assayed in a Cobas Mira Plus analyser (ABX Diagnostics, Switzerland) as previously reported [13]. Creatinine clearance, fractional excretion of sodium (FE_Na^+^_), and sodium balance were calculated as previously reported [13].

*ANP and cGMP Determination. *ANP levels in plasma samples as well as cGMP levels in urine and plasma samples were determined using commercial EIA kits following the manufacture's protocol from Phoenix Pharmaceuticals Inc. (USA) and R&D Systems (USA), respectively.

*Drugs*. The compounds PAN, zaprinast, and Sch-23390 were obtained from Sigma (USA).

### 2.3. Statistics

Results are expressed as means ± SE of values for the indicated number of determinations and were compared by one-way ANOVA followed by Student's *t*-test for unpaired comparisons. *P* < 0.05 was assumed to denote a significant difference.

## 3. Results

The sodium intake was similar in both PAN-NS and control rats throughout the study (Table 1). As previously described [13] PAN administration to Sprague-Dawley rats induced the development of high-range proteinuria and full-blown nephrotic syndrome with disturbed sodium balance (Table 1). In PAN-NS, the sodium balance profile is biphasic: on day 7 after injection, the PAN-NS rats compared with control rats exhibited a reduced FE_Na^+^_ accompanied with marked ascites accumulation, whereas on day 14 after injection the PAN-NS rats presented an increased FE_Na^+^_ accompanied with ascites of much smaller magnitude (Table 1). Plasma levels of sodium and potassium were similar in the two groups either on day 7 or day 14 (Table 1). Plasma levels of creatinine were higher in PAN-NS than in control rats both on days 7 and 14. This was accompanied by decreased creatinine clearance values in PAN-NS rats both on days 7 and 14 (Table 1). Plasma protein concentration was reduced in PAN-NS rats on day 7, but not on day 14 (Table 1).

**Figure 1:**
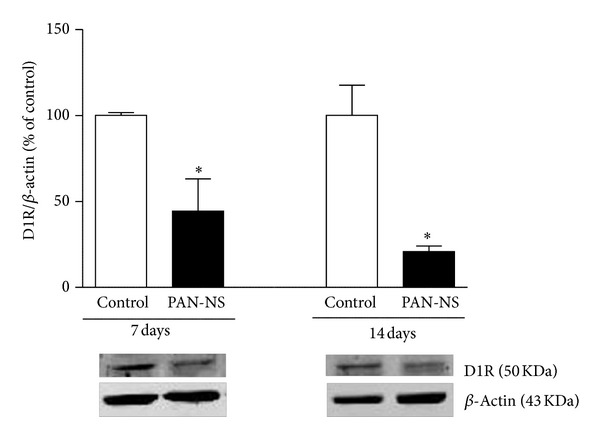
Protein expression of D1R in renal cortex of control and PAN-NS rats, seven and fourteen days after injection. The intensity of D1R bands (50 KDa) was quantified after normalization for *β*-actin protein expression (43 KDa) and is expressed as percentage of control. Bars represent means of 4 to 5 experiments per group and error bars represent SE. *P < 0.05, significantly different from values in control rats.

D1R protein expression was detected by western blotting in renal cortex as a 50 KDa molecular size band (Figure 1). The bands were quantified and normalized for *β*-actin protein (~43 KDa). In renal cortex of PAN-NS rats, D1R protein expression was markedly lower than that in control rats, 7 and 14 days after injection (~50% and ~80%, resp.) (Figure 1).

**Figure 2:**
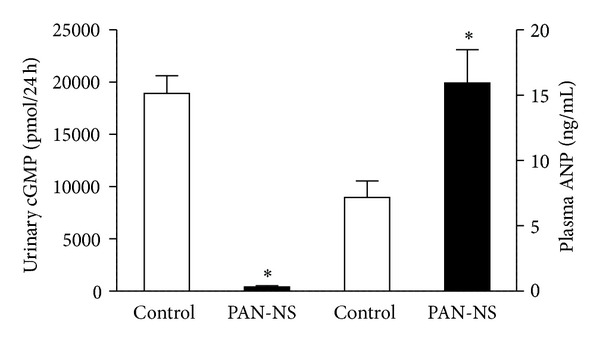
Plasma ANP levels and urinary cGMP excretion in control and PAN-NS rats, seven days after injection. Bars represent means of 5 experiments per group and error bars represent SE. *P < 0.05, significantly different from values in control rats.

On day 7 after PAN injection, sodium retention was accompanied by a twofold increase in plasma ANP levels and by a 98% reduction in urinary cGMP levels, compared with control rats (Figure 2).

**Figure 3:**
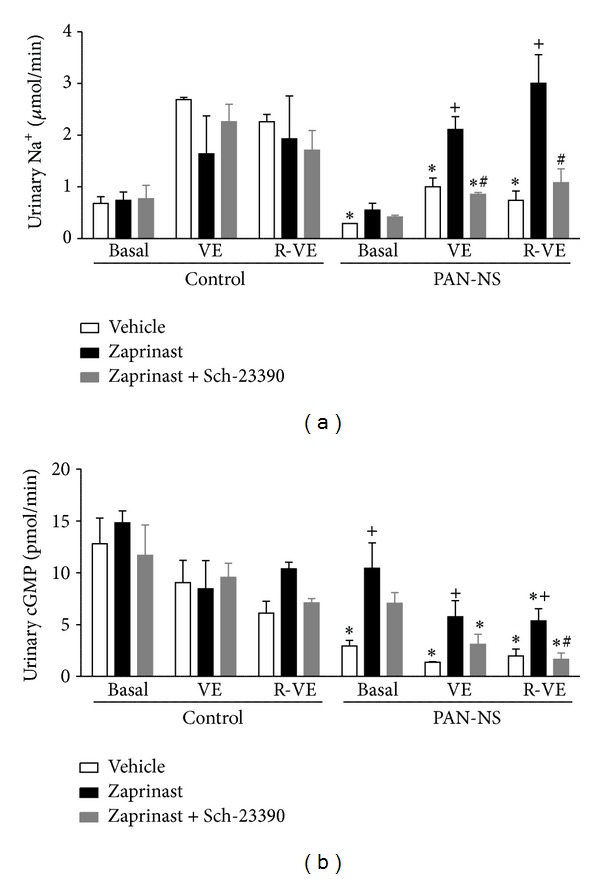
Urinary excretion of sodium (a) and cGMP (b) in control and PAN-NS rats before (*t* = 0–120 min, Basal), during (*t* = 120–150 min, VE), and after (*t* = 150–180 min, R-VE) volume expansion (VE), seven days after injection. Bars represent means of 3 to 4 experiments per group and error bars represent SE. *P < 0.05, significantly different from control rats within the same treatment group; ^+^ *P* < 0.05, significantly different from vehicle treatment. ^#^ *P* < 0.05, significantly different from zaprinast treatment.

The urinary excretion of sodium and cGMP before, during and after volume expansion in control and PAN-NS rats administered with vehicle (V), zaprinast (Z) and zaprinast plus Sch-23390 (ZS), is depicted in Figures 3(a) and 3(b). During all experimental periods, the urinary excretion of sodium in control rats was not altered by zaprinast or Sch-23390 infusions. PAN-NS V rats presented reduced urinary sodium excretion in comparison to control V animals, during all perfusion periods. Zaprinast infusion in PAN-NS rats was accompanied by a marked increase in urinary sodium excretion, which was more pronounced during volume expansion (VE) and recovery (R-VE) periods. This resulted in that urinary sodium excretion in PAN-NS Z rats did not differ from that observed in corresponding control animals throughout the study. Interestingly, when PAN-NS rats were infused with zaprinast plus Sch-23390, natriuresis was abolished and it did not differ from that observed in PAN-NS V group, throughout the study.

During all infusion periods, the urinary excretion of cGMP in control rats was not altered by zaprinast or Sch-23390 infusion. PAN-NS V rats presented reduced urinary cGMP excretion in comparison to control V animals. Zaprinast infusion in PAN-NS rats was accompanied by a marked increase in urinary cGMP excretion, during all infusion periods. This resulted in that urinary cGMP excretion in PAN-NS Z rats did not differ from that observed in corresponding controls during basal and VE periods. Interestingly, when PAN-NS rats were infused with D1R antagonist Sch-23390 together with zaprinast, the increase in cGMP urinary levels was markedly attenuated. Hence the urinary levels of cGMP in PAN-NS rats infused with zaprinast plus Sch-23390 did not differ from those observed in PAN-NS V group, throughout the study.

**Figure 4:**
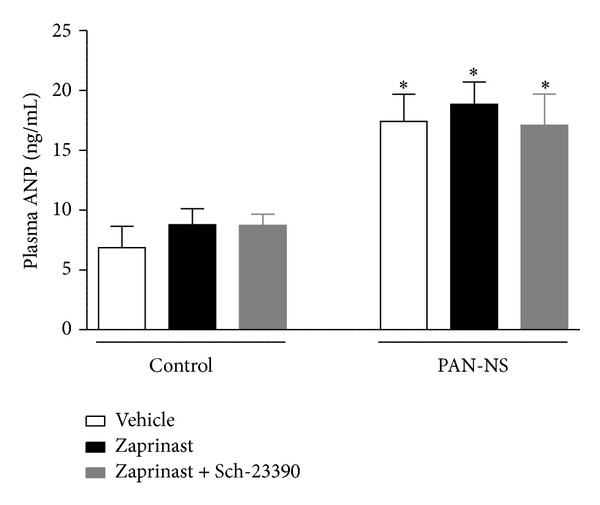
Plasma ANP levels in control and PAN-NS rats, seven days after injection. Bars represent means of 3 to 4 experiments per group and error bars represent SE. *P < 0.05, significantly different from control rats within the same treatment group.

Plasma ANP levels in control and PAN-NS rats at the end of the VE experiments were threefold higher in PAN-NS groups than those in the corresponding control groups (Figure 4). In addition, after infusion either with zaprinast or zaprinast plus Sch-23390, plasma ANP levels did not differ between control and PAN-NS rats.

**Figure 5:**
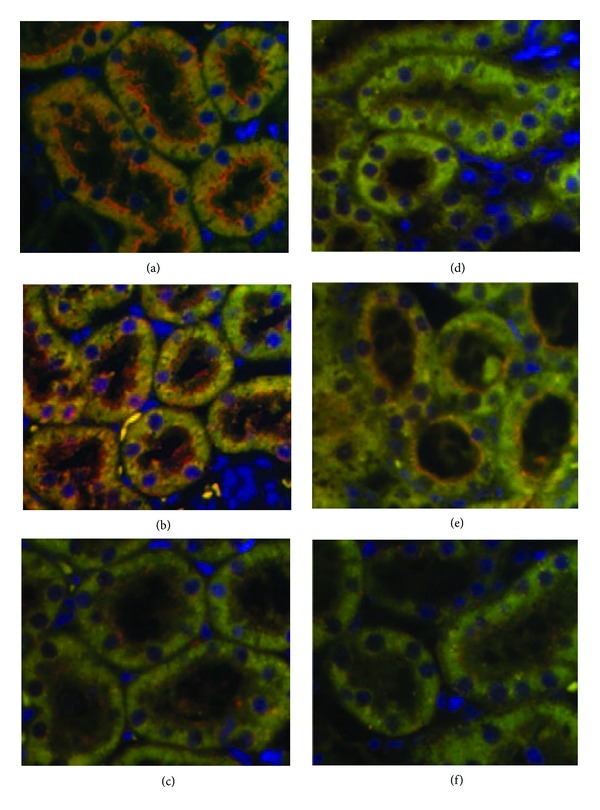
Localization of D1R in renal tubules of (a) control rats treated with vehicle, (b) control rats treated with zaprinast, (c) control rats treated with zaprinast plus Sch-23390, (d) PAN-NS rats treated with vehicle, (e) PAN-NS rats treated with zaprinast, and (f) PAN-NS rats treated with zaprinast plus Sch-23390, after volume expansion protocol. Double-immunofluorescence staining visualized by confocal microscopy was performed with polyclonal anti-D1R (red) antibody and monoclonal anti-gp330-megalin (green), colocalization (orange), and Hoechst 33258 stained for nuclei (blue). The images shown are representative of at least six images from 3 to 4 animals. Magnification 200x.

The effects of zaprinast plus Sch-23390 on D1R localization was examined in control and PAN-NS groups using double-immunofluorescence staining of renal tubules by confocal microscopy technique (Figures 5(a)–5(f)). As can be observed, D1R immunostaining was detected on the apical plasma membrane of renal tubule cells from vehicle-infused control animals (Figure 5(a)). By contrast, D1R immunostaining was absent on the apical plasma membrane of renal tubule cells from vehicle-infused PAN-NS rats (Figure 5(d)). After infusion of PDE5 inhibitor, zaprinast, D1R immunostaining in control group was unaltered. Interestingly, D1R immunostaining was detected on the plasma membrane of renal tubule cells from zaprinast-infused PAN-NS rats with a distribution similar to that of the corresponding control group, thus suggesting a cGMP-induced translocation of D1R toward the apical plasma membrane in zaprinast-treated PAN-NS rats (Figure 5(e)). After infusion of the D1R antagonist, Sch-23390, D1R immunostaining on the apical plasma membrane of renal tubule cells disappeared in both control and PAN-NS rats (Figures 5(c) and 5(f)).

## 4. Discussion

Our study provides new insights on the intrarenal alterations responsible for sodium retention of nephrotic edema induced by PAN administration in rats. Firstly, the abundance of D1R, their specific activity, and subcellular localization are altered in renal tubules of PAN-NS rats. Secondly, the natriuretic response observed in PAN-NS rats during infusion of the PDE5 inhibitor, zaprinast, was accompanied by an increased translocation of D1R from the cytosol of the renal tubular cells towards the plasma membrane [18]. These data, when viewed collectively with the finding that, in PAN-NS rats, the natriuretic response resulting from inhibition of enhanced cellular cGMP catabolism was blunted when the D1R antagonist Sch-23390 was simultaneously administered, support the notion that renal dopamine and D1R contribute to the *in vivo* natriuretic effect associated with corrected resistance to ANP in experimental NS.

As previously reported [13], PAN administration to Sprague-Dawley rats induced the development of high-range proteinuria and full-blown NS with disturbed sodium balance. In PAN-NS, sodium retention and proteinuria follow independent courses. In addition, ascites is biphasic and appears only when proteinuria is accompanied by marked sodium retention. These data support the view that the reduction of urinary sodium excretion in PAN-NS occurs independently of the development of proteinuria and may be attributed to a tubular disorder. 

Given that in PAN-NS sodium homeostasis follows a biphasic curve, animals were sacrificed on days 7 and 14, because those are the days that better correspond to the phase of sodium retention (day 7) and to the phase of sodium excretion (day 14).

A blunted renal dopaminergic system activity was previously described in PAN-NS [13]. This was evidenced by decreases in renal aromatic L-amino acid decarboxylase activity and urine dopamine output, going along with an increase in Na^+^, K^+^-ATPase activity in renal tubules. In addition, inability of D1R agonist fenoldopam to induce natriuresis was observed in a previous study performed by our group in PAN-NS during the period of maximum sodium retention and ascites accumulation [13]. The present data provide new insights into the decreased renal dopamine system activity in PAN-NS by showing that the blunted natriuretic response to fenoldopam is related with a decreased D1R protein expression per se and with a reduced abundance of active receptors in the apical plasma membrane of renal tubular cells from PAN-NS rats. Additionally, as D1R expression is reduced not only during the period of maximum sodium retention but also during the period of sodium excretion, one can suggest that a molecular mechanism other than D1R activation via renal dopamine is responsible for the sodium excretion observed in PAN-NS rats.

Rapid intravenous infusion of normal saline, equal to 5% rat's weight, caused an immediate, large diuresis and natriuresis in normal rats, whereas animals with PAN-NS exhibited a significantly reduced natriuretic response. The reduced excretion of cGMP in nephrotic rats, despite elevated plasma ANP levels, provides a means to link the blunted volume expansion natriuresis to the previously demonstrated resistance to infused ANP through a cellular defect in the accumulation of its intracellular second messenger cGMP in target tissues [6, 7]. As expected, nephrotic rats infused with the PDE5 inhibitor, zaprinast, exhibited a natriuretic response that was indistinguishable from the response by corresponding control rats. The zaprinast infusion also normalized the urine cGMP excretion in nephrotic rats, thus supporting an abnormality in cGMP metabolism in responsive tissues in PAN-NS, related to heightened activity of cGMP-PDE. The finding that the infusion of D1R antagonist Sch-23390 together with zaprinast abolished the natriuretic response induced by the PDE5 inhibitor alone provides evidence that the renal dopaminergic system contributes to the natriuretic response associated with the corrected resistance to natriuretic peptide in PAN-NS. This hypothesis was further reinforced by the observation that zaprinast-induced natriuresis in PAN-NS rats was associated with translocation of D1R to the apical plasma membrane of proximal tubular cells, assessed by means of double-immunofluorescence staining and confocal microscopy. 

When the D1R antagonist was infused together with zaprinast, the increase in cGMP excretion observed during infusion of zaprinast alone was not observed. This finding suggests that, in PAN-NS, the D1R antagonist or the absence of dopamine binding to its receptor may alter ANP receptors before signal transduction. There is still little information regarding natriuretic peptides receptors (NPRs) status in NS. Although it was suggested that the abnormality responsible for ANP resistance in NS lies mainly at postreceptor locus [6, 7] recent studies suggested that a downregulation of renal NPR-A may also contribute to ANP resistance in PAN-NS during the period of highest sodium retention and ascites accumulation [19]. It is clear that additional studies are required to elucidate the contribution of the renal dopamine system to ANP resistance in NS, namely, in relation to changes in NPR status.

We herein suggest that the dysfunction of renal dopaminergic and natriuretic peptide systems interacts strongly with and contributes to sodium retention in experimental NS. The molecular mechanisms by which these two systems interact still need to be clarified. Correa and coworkers [20] have demonstrated that ANP induces DA uptake in renal tubular cells by activation of the natriuretic peptide receptor-type A (NPR-A) coupled to guanylate cyclase with the involvement of cGMP and the protein kinase, PKG, in this signaling pathway. Moreover, Eklöf and coworkers have demonstrated that mice lacking the gene that encodes DARPP-32, an intracellular third messenger of dopamine, develop hypertension and present no ability to induce ANP-mediated natriuresis [21]. The signaling pathways of both natriuretic hormones, dopamine and ANP, involve the PKA and PKG that after being activated by their second intracellular messengers, cAMP and cGMP, respectively, directly phosphorylate and inactivate Na^+^, K^+^-ATPase [20]. Both these protein kinases are involved in DARPP-32 phosphorylation, in which the latter leads to inactivation of Na^+^, K^+^-ATPase [22]. Thus, DARPP-32 could play an important role in the intricate process that links ANP and renal dopaminergic system activities and modulation. Further studies are required to address these questions.

In summary, we have found that the intrarenal sodium handling abnormality in PAN-NS related to ANP resistance is associated with decreased abundance and altered subcellular localization of D1R in renal tubules, whereas corrected resistance to natriuretic peptides in PAN-NS resulting from inhibition of cGMP-PDE5 is accompanied by recruitment of D1R to the plasma membrane of renal tubules. The finding that blockade of D1R markedly attenuated the natriuretic response induced by the PDE5 inhibitor suggests the possibility that, in PAN-NS, dopamine and D1R contribute importantly to the *in vivo* natriuretic effect of ANP. Further study of the synergistic effects of ANP and dopamine should provide new insight into the basis for pathological sodium retention in NS.

## Figures and Tables

**Table 1:** Body weight, metabolic balance, and renal function in control and PAN-NS rats, seven and fourteen days after injection.

	Day 7		Day 14	
	Control	PAN-NS	Control	PAN-NS
Body weight (g)	161 ± 2	190 ± 4*	241 ± 2	234 ± 5
Food intake (g/24 h)	14.08 ± 0.23	14.85 ± 0.29	15.38 ± 0.53	19.29 ± 2.33
Fluid intake (mL/24 h)	19.82 ± 0.86	31.63 ± 1.38*	21.88 ± 0.69	21.86 ± 4.37
P Na^+^ (mmol/L)	144 ± 1	143 ± 1	135 ± 3	135 ± 1
P K^+^ (mmol/L)	5.50 ± 0.37	6.13 ± 0.77	5.23 ± 0.25	5.80 ± 0.30
P creatinine (mg/L)	2.84 ± 0.09	5.04 ± 0.39*	2.20 ± 0.20	3.20 ± 0.30*
P proteins (g/L)	47.84 ± 0.41	35.98 ± 3.02*	49.42 ± 1.55	48.98 ± 2.98
C creatinine (mL/min)	3.91 ± 0.39	2.25 ± 0.28*	4.51 ± 1.08	2.24 ± 0.32*
Na^+^ intake (mmol/24 h)	1.16 ± 0.02	1.20 ± 0.02	1.31 ± 0.05	1.57 ± 0.28
Ascites (g)	0.61 ± 0.08	14.31 ± 2.46*	0.60 ± 0.10	1.30 ± 0.30*
Na^+^ balance (mmol/24 h)	0.15 ± 0.06	0.89 ± 0.07*	0.18 ± 0.13	−0.61 ± 0.38*
FE_Na^+^_ (%)	0.32 ± 0.02	0.05 ± 0.01*	0.17 ± 0.03	0.53 ± 0.14*
U Na^+ ^(mmol/24 h)	1.02 ± 0.06	0.32 ± 0.05*	1.13 ± 0.13	2.00 ± 0.24
U K^+ ^(mmol/24 h)	1.30 ± 0.11	1.82 ± 0.16*	1.37 ± 0.05	1.92 ± 0.17*
U proteins (mg/24 h)	30 ± 4	298 ± 40*	38 ± 7	438 ± 47*
U creatinine (mmol/24 h)	0.046 ± 0.001	0.049 ± 0.004	0.092 ± 0.009	0.085 ± 0.009

Values are means ± SE; *n* = 4 to 5 experiments per group. U: urinary; FE: fractional excretion; P: plasma; and C creatinine: creatinine clearance. *P < 0.05, significantly different from corresponding values in control rats.
